# Comparative Study on Pharmacokinetics of Four Active Compounds in Rat Plasma after Oral Administration of Raw and Wine Processed Chuanxiong Rhizoma

**DOI:** 10.3390/molecules25010093

**Published:** 2019-12-25

**Authors:** Yan Ning, Ke Pei, Gang Cao, Hao Cai, Xiao Liu, Lilong Cao, Shuosheng Zhang, Baochang Cai

**Affiliations:** 1 Shanxi Engineering Laboratory of Modern Chinese Medicine, College of Traditional Chinese Medicine, Shanxi University of Chinese Medicine, Taiyuan 030619, China; ny18434375920@163.com (Y.N.); C178798032@126.com (L.C.); zhangshuosheng@aliyun.com (S.Z.); 2 Research Center of TCM Processing Technology, Zhejiang Chinese Medical University, Hangzhou 310053, China; caogang33@163.com; 3 School of Pharmacy, Nanjing University of Chinese Medicine, Nanjing 210023, China; liuxiao04_0@163.com (X.L.); bccai@126.com (B.C.)

**Keywords:** Chuanxiong Rhizoma, UHPLC-MS/MS, wine processed, pharmacokinetics

## Abstract

In Chinese medicine, the effect of promoting blood circulation and removing stasis could be enhanced after Chuanxiong Rhizoma is processed by wine. However, the relevant mechanism remains unclear. In this manuscript, a rapid and sensitive quantification method employing ultrahigh performance liquid chromatography-tandem mass spectrometry (UHPLC-MS/MS) was established and validated to simultaneously determine butylidenephthalide, ligustilide, senkyunolide A and ferulic acid in rat plasma after oral administration of raw Chuanxiong Rhizoma (RCR) and wine-processed Chuanxiong Rhizoma (WCR) respectively. All analytes were extracted from plasma by proteins precipitation with methanol. Chromatographic separation was carried out on a Hypersil GOLD C_18_ column by using a gradient mobile phase system of acetonitrile and water with 0.01% formic acid, the flow rate was 0.3 mL/min. For exact mass detecting, quick switching mode was used, positive and negative ions could be detected in one injection. The pharmacokinetic profiles of four components in the two groups were evaluated and compared. The results showed that, compared to the RCR group, the V_d_ and AUC_0→t_ values of four active compounds were increased and decreased respectively in WCR group, which revealed the effect of wine processing to Chuanxiong Rhizoma: the stronger the effect, the wider the distribution.

## 1. Introduction

Chuanxiong Rhizoma, the dried rhizome of *Ligusticum chuanxiong* Hort., is one of the commonly used traditional Chinese medicines (TCM). It has been claimed to have potent functions such as promoting blood circulation and dispelling wind. Chuanxiong Rhizoma has also been used to relieve pain caused by blood stasis and qi stagnation, containing chest pain, irregular menstruation, dysmenorrhea, amenorrhea, postpartum abdominal pain and headache in Chinese [1]. It is reported that Chuanxiong Rhizoma exhibits a variety of pharmacological activities, including protecting cardiovascular and cerebrovascular system [2,3,4,5], anti-oxidation [6,7,8], neuroprotection [9], anti-fibrosis [10], anti-nociception [11], anti-inflammation [12], and anti-neoplastic activity [13].

**Figure 1:**
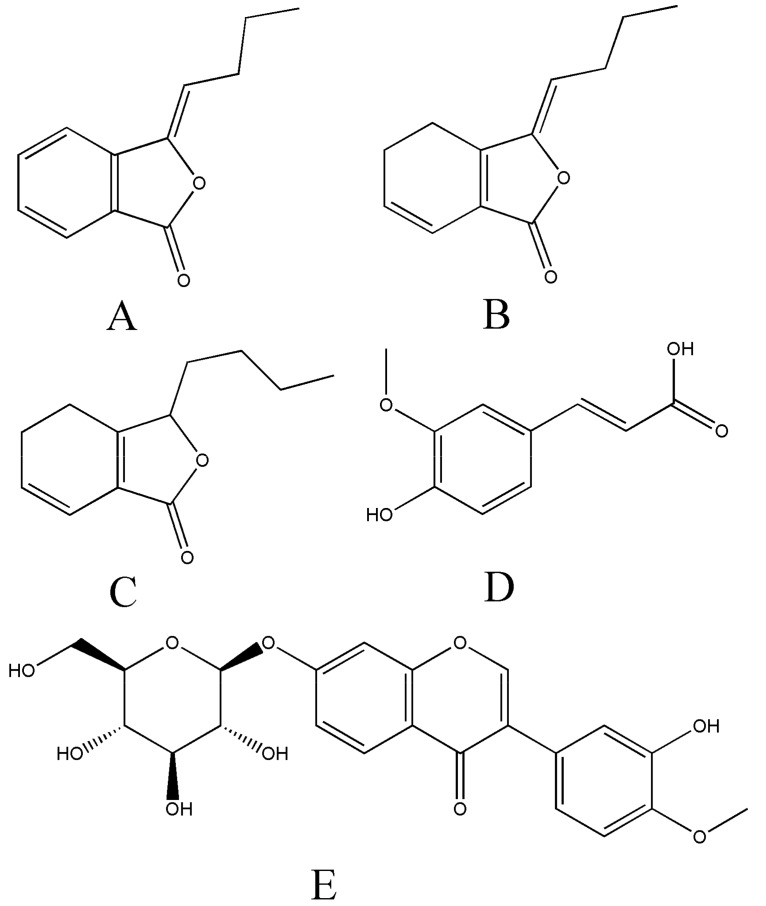
Chemical structures of butylidenephthalide (**A**), ligustilide (**B**), senkyunolide A (**C**), ferulic acid (**D**) and calycosin-7-*O*-*β-*d-glucoside (**E**, IS).

The chemical constituents of Chuanxiong Rhizoma can be roughly divided into several categories, like phenolic acids, alkaloids, and phthalides [14]. Our preliminary study has revealed that butylidenephthalide, ligustilide and senkyunolide A were the main components of phthalides in volatile oil of Chuanxiong Rhizoma. In the Chinese Pharmacopoeia 2015 Volume 1, ferulic acid was selected to control the quality of Chuanxiong Rhizoma. Besides, our previous research showed that, by comparing to raw Chuanxiong Rhizoma (RCR), the contents of butylidenephthalide, ligustilide senkyunolide A and ferulic acid were increased in wine-processed Chuanxiong Rhizoma (WCR). Additionally, these four components have many biological properties and pharmacological functions [15,16,17,18]. Therefore, they were selected as the target analytes in our experiments. Their chemical structures were shown in Figure 1.

Prior to clinical practice, the harvested crude drugs were often processed by TCM processing technology, named Pao Zhi, such as cleaning, cutting, stir-frying, steaming and calcining. Only in this way can crude drugs convert into readily utilized herbal forms called decoction pieces (Yinpian), that could be used for prescription, clinical decoctions, and manufacturing [19,20]. Various processing methods of Chuanxiong Rhizoma were recorded in ancient documents, while the wine stir-frying was considered to be the most common way, which can enhance efficiency including activating blood circulation and removing blood stasis [21,22]. However, the relevant mechanism remains unclear. Many researchers think that the processing of adjuvant-the rice wine could increase the dissolution of active compounds, and this is the reason why WCR has a stronger effect of activating blood circulation. In our opinion, increasing the dissolution may or may not be the case. The serum pharmaceutical chemistry suggested that the components absorbed into blood are more likely to be the active ingredients, no matter whatever changed in vitro [23,24]. Hence, in order to further understand the mechanism of wine processing, it is necessary to compare the pharmacokinetic profile differences between RCR and WCR. In our manuscript, an improved UHPLC-Q-Exactive-Orbitrap/mass spectrometry (MS) method was developed and validated for the simultaneous determination of four compounds (butylidenephthalide, ligustilide, senkyunolide A and ferulic acid) in rat plasma after oral administration of RCR and WCR. The pharmacokinetic parameters of the above four compounds were calculated and compared. The established approach provides the scientific basis for the influence of wine processing on the efficacy of Chuanxiong Rhizoma in vivo.

Q Exactive Focus Orbitrap mass system equipped with high selective performance of quadrupole and high resolving power of Orbitrap has advantages of superior accuracy (10^−7^–10^−6^), high resolution (up to 1,000,000) and excellent sensitivity [25]. It can provide multiple scan patterns, like full scan, parallel reaction monitoring, and selected ion monitoring. Its full mass scan mode could display with up to 5 decimal places of resolution, and the secondary fragments ion could be provided when dd-MS^2^ (data-dependent MS^2^) function module was added. Furthermore, Q Exactive Focus Orbitrap has a fast positive and negative ion switching function, which can simultaneously detect positive and negative ions when analyzing samples. Therefore, it is a very effective way to realize simultaneously qualitative and quantitative analysis of multiple components [26,27,28,29]. In this study, full mass scan mode equipped with quick switching and dd-MS^2^ modules was used, both positive and negative ions (butylidenephthalide, ligustilide and senkyunolide A were detected in positive ion mode, ferulic acid was detected in negative ions mode) were detected in one injection. Moreover, exact molecular mass was used for quantification to ensure the specificity and accuracy of the determination. The established method was proved to be time and effort saving as well as reliable.

Besides, the innovation of this work was also reflected in that, we have revealed the possible mechanism of WCR possessing enhanced effect from a new perspective: the stronger the effect, the wider the distribution. Even though the contents of active compounds in WCR were increased significantly, the AUC values (both AUC_0→t_ and AUC_0→∞_) and *C_max_* values were still decreased after the oral administration of WCR. Instead, V_d_ values of four compounds in the WCR group were all increased significantly. Hence, promoting the blood circulation and increasing the distribution could be the influence of wine-processing on the efficacy of Chuanxiong Rhizoma.

## 2. Results and Discussion

### 2.1. Optimization of Chromatographic and Mass Spectrum Conditions

To improve the resolution and intensity of chromatographic peaks, various compositions of the mobile phase were tested. As a result, acetonitrile (A) and 0.01% (*v/v*) aqueous formic acid (B) were selected as the mobile phases. The flow rate was eventually determined at 0.3 mL/min, and the column temperature was held at 40 °C throughout the period of analysis.

**Figure 2:**
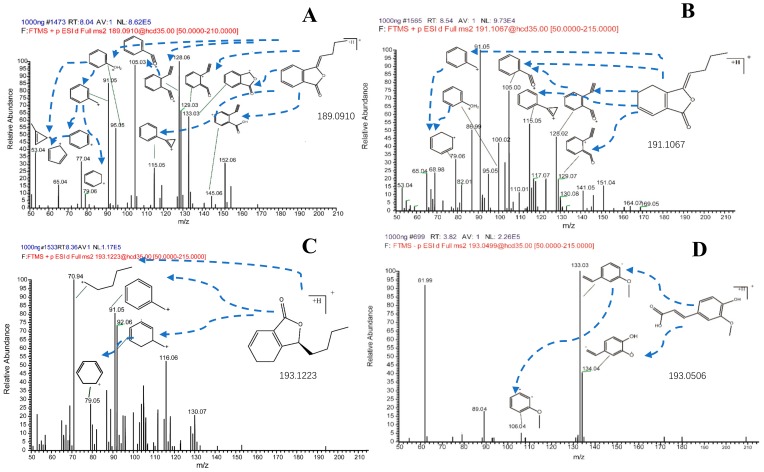
The fragment mass spectrometry (MS/MS spectrometry) of butylidenephthalide (**A**), ligustilide (**B**), senkyunolide A (**C**) and ferulic acid (**D**).

In order to obtain better responses, some ion source parameters such as spray voltage, flow rate of sheath gas and capillary temperature were also optimized. In comparison with positive ion mode, the response of butylidenephthalide, ligustilide, senkyunolide A in negative ion mode was weak or even undetectable, and the intensity of [M + H]^+^ was better than other adducts. However, for ferulic acid, the response in negative ion mode was better, and the intensity of [M − H]^−^ was better than other adducts. Therefore, *m/z* 189.09105, 191.10664, 193.12244, 193.04997 for butylidenephthalide, ligustilide, senkyunolide A and ferulic acid respectively were determined in the full mass scan. The product mass spectra of the analytes were presented in Figure 2. The measured and theoretical masses of four analytes were showed in Table 1. The mass error of each analyte was less than 5 ppm by using the established method. Both exact molecular mass detecting and secondary fragment assigning could ensure the specificity and accuracy of the determination.

### 2.2. The Quantitative Analysis of Four Compounds in Oral Liquid Medicine of RCR and WCR

**Figure 3:**
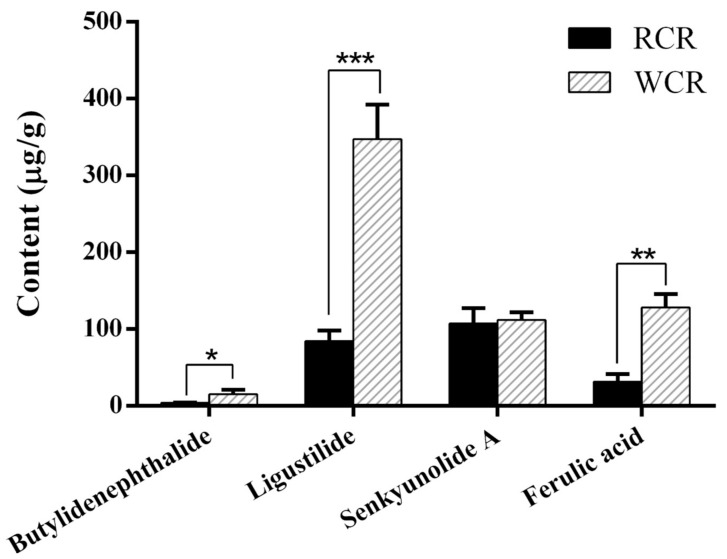
The contents of butylidenephthalide, ligustilide, senkyunolide A and ferulic acid in oral liquid medicine of raw Chuanxiong Rhizoma (RCR) and wine-processed Chuanxiong Rhizoma (WCR) in vitro. (* *P* < 0.05, ** *P* < 0.01, *** *P* < 0.0001).

The contents of butylidenephthalide, ligustilide, senkyunolide A and ferulic acid in oral liquid medicine of RCR and WCR have been determined firstly in our study, there were 4.05, 84.46, 107.12 and 31.30 µg/g respectively in oral liquid medicine of RCR, while 15.21, 347.48, 111.91 and 128.13 µg/g respectively in oral liquid medicine of WCR. The results showed that the progress of wine processing can cause a significant increase (about a three-fold increase) in the butylidenephthalide, ligustilide, and ferulic acid content of WCR. The contents of butylidenephthalide, ligustilide, senkyunolide A and ferulic acid in oral liquid medicine of RCR and WCR were shown in Figure 3.

### 2.3. Sample Preparation

Protein precipitation, solid-phase extraction, and liquid phase extraction were all tested for sample extraction. The results showed that the recovery obtained from liquid phase extraction could not satisfy the requirement of pharmacokinetic analysis. Compared with protein precipitation, it was expensive for a larger number of solid-phase extraction cartridges when solid-phase extraction method was used, and extraction recovery was not obviously improved. Therefore, protein precipitation with methanol was finally selected as the ultimate method of sample extraction owing to its advantages of saving time, simple operation and high extraction efficiency.

### 2.4. Method Validation

#### 2.4.1. Specificity

**Figure 4:**
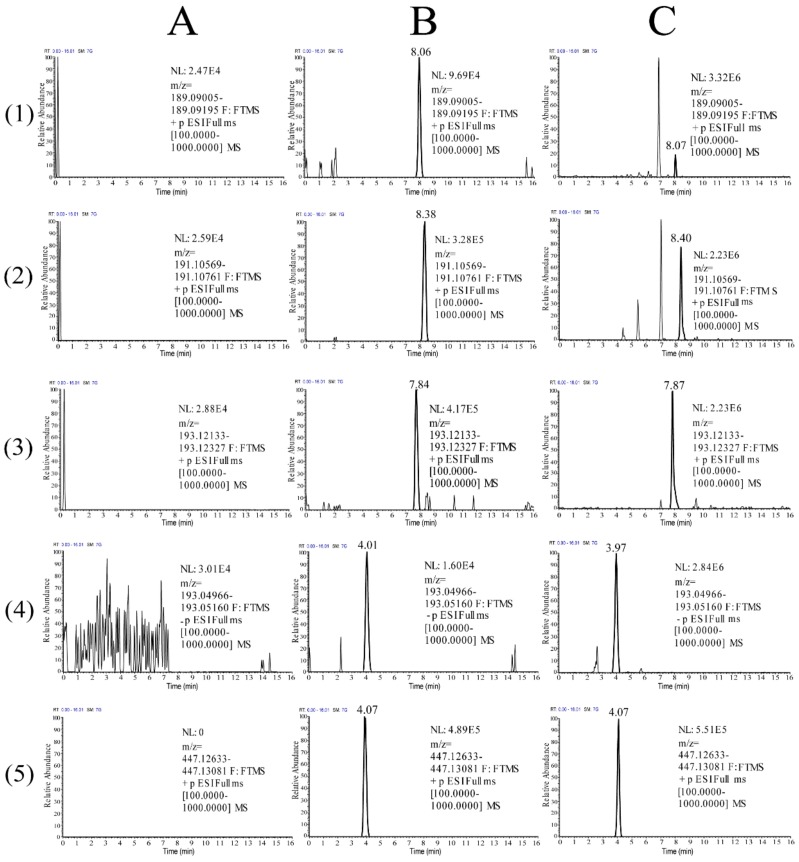
Representative chromatograms of butylidenephthalide (**1**), ligustilide (**2**), senkyunolide A (**3**), ferulic acid (**4**) and calycosin-7-*O*-*β*-d-glucoside (IS) (**5**) in rat plasmas: (**A**) blank plasma samples; (**B**) blank plasma samples spiked with butylidenephthalide, ligustilide, senkyunolide A, ferulic acid at linearity and lower limit of quantitation (LLOQ) and IS; (**C**) plasma samples obtained at 1 h after oral administration of RCR at a dose of 24.01 g/kg.

The representative chromatograms of blank plasma samples, blank plasma samples spiked with standard solutions containing target analytes at linearity and lower limit of quantitation (LLOQ) and IS and rat plasma samples collected at 1 h after oral administration of RCR at a dose of 24.01 g/kg were presented in Figure 4. As can be seen from the figure, the retention times were approximately.

8.05, 8.38, 7.86, 3.99 and 4.05 min for butylidenephthalide, ligustilide, senkyunolide A, ferulic acid and the IS, respectively. At the same time, the endogenous substances as well as metabolites in plasma had no interference with the determination of analytes and IS.

#### 2.4.2. Linearity and LLOQ

The calibration curves of the four analytes were linear in the ranges of 3.54–1415 ng/mL for butylidenephthalide, 2.61–1044 ng/mL for ligustilide, 3.28–1310 ng/mL for senkyunolide A and 3.34–13350 ng/mL for ferulic acid, respectively. Besides, the correlation coefficient of each standard curve was greater than 0.99. More specifically, the regression equations and correlation coefficients (r) of the calibration curves were y = 2.3257x + 0.2319, r = 0.999 for butylidenephthalide, y = 7.7234x − 0.1415, r = 0.999 for ligustilide and y = 2.8285x + 0.16672, r = 0.998 for senkyunolide A. For ferulic acid, according to the results of preliminary test, there was a wide range between low and high plasma concentrations, to make the determination more accurate, two standard curves were established for ferulic acid. They were y = 0.8378x + 0.0998, r = 0.996 (3.34 ng/mL < x < 133.50 ng/mL) and y = 0.7965x + 1.2854, r = 0.999 (133.50 ng/mL < x < 13350 ng/mL).

The LLOQ of butylidenephthalide, ligustilide, senkyunolide A, ferulic acid was 3.54, 2.61, 3.28, and 3.34 ng/mL, respectively. The precision (RSD) were 8.89%, 11.28%, 13.26%, and 10.86%, respectively (all below 20%) and the accuracy (RE) were 4.09%, −4.80%, 10.43%, and −7.91%, respectively (all within ± 20%).

#### 2.4.3. Precision and Accuracy

The precision and accuracy results were shown in Table 2. The precision results of intra- and inter-day of four analytes ranged from 5.17% to 12.88%, while the accuracy results ranged from −8.14% to 11.70%, all within the acceptable range. The results have shown that the method was reliable and reproducible.

#### 2.4.4. Extraction Recovery and Matrix Effect

The extraction recoveries of butylidenephthalide, ligustilide, senkyunolide A, and ferulic acid from QC samples at three concentrations levels were between 92.61% and 110.78%, while the mean extraction recovery of IS was 104.57% (Table 2). The recovery results demonstrated the sample processing method used in this experiment can effectively extract analytes from biological samples. The calculated values of the matrix effect were between 91.23% and 105.63% (Table 2), which suggested there was no obvious matrix effect for this method when four analytes were determined.

#### 2.4.5. Stability

The stability results under various conditions were shown in Table 3. All values were in acceptable range, which fully illustrated that all analytes in rat plasma were stable for 4 h at room temperature, for 60 days at −20 °C, after three freeze-thaw cycles, and for 24 h in autosampler at 4 °C after pretreatment.

### 2.5. Pharmacokinetic Application and Discussion

**Figure 5:**
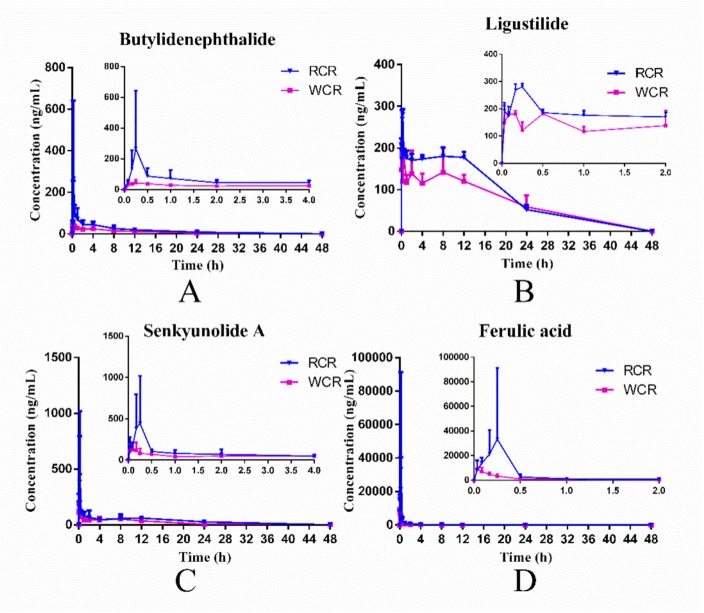
Mean (*n* = 6) plasma concentration–time curves of butylidenephthalide (**A**), ligustilide (**B**), senkyunolide A (**C**) and ferulic acid (**D**) after oral administration of RCR and WCR.

The validated UHPLC-Q-Exactive-Orbitrap/MS method was successfully applied to pharmacokinetic study of four components in rat plasma after oral administration of RCR and WCR respectively. The concentration-time curves of butylidenephthalide, ligustilide, senkyunolide A and ferulic acid were established and shown in Figure 5. The pharmacokinetic parameters of four compounds were summarized in Table 4.

As shown in Table 4 and Figure 5, compared to RCR, the AUC values (both AUC_0→t_ and AUC_0→∞_) and *C_max_* values of butylidenephthalide, ligustilide, senkyunolide A and ferulic acid in WCR group were decreased significantly, which were completely opposite results to what we initially expected. Because, the butylidenephthalide, ligustilide, and ferulic acid content in oral liquid medicine of WCR were roughly four times higher than RCR. Therefore, the AUC values (both AUC_0→t_ and AUC_0→∞_) and *C_max_* values of the WCR group were supposedly higher than RCR group. What is interesting, however, is that these values of butylidenephthalide, ligustilide, senkyunolide A and ferulic acid in WCR group were all decreased significantly. Besides, the V_d_ values of four compounds of WCR group showed a contrary tendency with the changes of AUC and *C_max_* values. The results remind us that processing with rice wine could increase the distribution of four compounds, so a larger amount of butylidenephthalide, ligustilide, senkyunolide A and ferulic acid may exist in organs. Moreover, the CL value of butylidenephthalide, ligustilide, senkyunolide A and ferulic acid in the WCR group were all increased significantly, which was also the supporting evidence of our reasoning above.

According to our study results, the possible reasons of WCR group with increasing V_d_ values was that after processed by rice wine, the promoting blood circulation function of Chuanxiong Rhizoma was enhanced, which could improve the exchange of chemicals between the blood and the tissues, so the distributions of four compounds were increased. This experiment result and theoretical analysis agrees well with the theory of traditional Chinese medicine that the wine processing method could promote blood circulation. However, other possible mechanisms may also exist, such as the method of wine processing could improve vascular permeability, improve microcirculation and so on, which still needs further research.

## 3. Materials and Methods 

### 3.1. Chemicals and Reagents

Butylidenephthalide, ligustilide, senkyunolide A and ferulic acid (purity > 98%) were purchased from Chengdu Herbpurify Co., Ltd. (Chengdu, China). Calycosin-7-*O*-*β*-d-glucoside (purity > 98%), used as internal standard (IS), was purchased from Shanghai Standard Technology Co., Ltd. (Shanghai, China). LC-MS grade acetonitrile and methanol were obtained from Thermo Fisher Technology Co., Ltd. (Fair Lawn, NJ, USA). Formic acid was of chromatographic grade from Tianjin Guangfu Fine Chemical Research Institute (Tianjin, China). The experimental yellow wine was supplied from Shaoxing Wuyue Brewing Co., Ltd. (Zhejiang, China). All other reagents were of analytical grade. Purified water was obtained from a Milli-Q system (Millipore, Bedford, MA, USA).

### 3.2. WCR Preparation

Chuanxiong Rhizoma used in the experiment was produced in Sichuan (it’s indigenous cultivating region), and authenticated by Prof. Shuosheng Zhang. An appropriate amount of yellow rice wine was added onto Chuanxiong Rhizoma decoction pieces, then the slices were turned over from time to time until the wine was absorbed thoroughly. The wine processed slices were stir-fried for 20 min at 100 °C after completely moistened. Finally, stir-fried slices were spread out and cooled at room temperature, then the WCR was obtained.

### 3.3. Oral Liquid Medicine of RCR and WCR Preparation

RCR (300 g) and WCR (300 g) were weighed and soaked in water (6:1, *v*/*w*) for 20 min respectively, then refluxed for 30 min and extracted twice. Finally, the extracts of RCR and WCR were filtered, combined, and concentrated to 1.20 g/mL respectively. The concentrated extracts were stored in a refrigerator at 4 °C before use.

### 3.4. Animals

Male adult Sprague-Dawley rats (220–250 g) were obtained from the Laboratory Animal Center of Shanxi Medical University (Shanxi, China). The rats were fed in an air-conditioned animal center with a natural light-dark cycle for a week, the temperature was maintained at 22–26 °C and the relative humidity was maintained at 60 ± 10%. All procedures involving the animal were in accordance with the Regulations of Experimental Animal Administration issued by the State Committee of Science and Technology of People’s Republic of China. It is worth noting that all rats were fasted with free access to water for 12 h prior to the experiment.

### 3.5. UHPLC-Q-Exactive-Orbitrap/MS Introduction

An Ultimate 3000 system (Thermo Fisher Scientific, Dionex, Sunnyvale, CA, USA) consisting of a binary pump (HPG-3400RS), an autosampler (WPS-3000), a column compartment (TCC-3000), and an online degasser was used for chromatography separation. Quantitative analysis was performed by using a quadrupole-orbitrap mass spectrometer equipped with an electrospray ionization (ESI) interface and an orbitrap mass analyzer (Thermo Fisher Scientific, San Jose, CA, USA).

#### 3.5.1. Liquid Chromatography

The separation of four compounds was executed by a linear gradient elution using a mobile phase consisting of acetonitrile (A) and 0.01% (*v/v*) aqueous formic acid (B). The gradient elution procedure was as follows: 0–0.5 min, 5% A–5% A; 0.5–1.5 min, 5% A–15% A; 1.5–4.5 min, 15% A–30% A; 4.5–7 min, 30% A–60% A; 7–11 min, 60% A–70% A; 11–12 min, 70% A–100% A; 12–13 min, 100% A–100% A; 13–13.5 min, 100% A–5% A; 13.5–16 min, 5% A–5% A. The flow rate during this period was always 0.3 mL/min and the injection volume was 5 µL. The retention time of butylidenephthalide, ligustilide, senkyunolide A and calycosin-7-*O*-*β*-d-glucoside (IS) in positive ionization mode was 8.05, 8.38, 7.86 and 4.05 min, respectively. Meanwhile, the retention time of ferulic acid in negative ionization mode was 3.99 min.

#### 3.5.2. Mass Spectrometer

The mass spectrometry conditions were optimized as follows: sheath gas flow rate, 40 arb; aux gas flow rate, 5 arb; sweep gas flow rate, 1 arb; spray voltage, 3.2 kV; capillary temperature, 320 °C; aux gas heater temperature, 350 °C; S-lens RF level, 50. A full MS scan with a mass range of *m/z* 100–1000 was used for compound detecting. Both positive and negative ions were scanned in a mode of fast switching between positive and negative ions under the condition of resolution 70,000 FWHM. The automatic gain control target and a maximum injection time were set at 2 × 10^5^ and 50 milliseconds respectively. Xcalibur 4.0 software was used for data processing and quantitative analyses.

### 3.6. Preparation of Standard and Quality Control Samples

Butylidenephthalide (2.83 mg), ligustilide (2.09 mg), senkyunolide A (2.62 mg) and ferulic acid (2.67 mg) were accurately weighed and dissolved in methanol to obtain primary stock solutions with concentrations of 0.28 mg/mL, 0.21 mg/mL, 0.26 mg/mL and 0.27 mg/mL respectively. Working solutions of the calibration curve were prepared by mixing and serial diluting each stock solution with methanol. In addition, IS stock solution was prepared by dissolving 2.43 mg of calycosin-7-*O*-*β*-d-glucoside in 10 mL methanol then further diluting to obtain a final standard solution of 607.50 ng/mL. All solutions were stored at 4 °C until analysis.

The standard plasma samples were prepared by spiking 10 µL of corresponding working solution into 100 µL blank rat plasma to achieve final concentration of 3.54, 7.08, 14.15, 70.75, 141.50, 707.50, 1415 ng/mL for butylidenephthalide; 2.61, 5.22, 10.44, 52.20, 104.40, 522, 1044 ng/mL for ligustilide; 3.28, 6.55, 13.10, 65.50, 131, 655, 1310 ng/mL for senkyunolide A and 3.34, 6.68, 13.35, 66.75, 133.50, 667.50, 1335, 13,350 ng/mL for ferulic acid.

The quality control (QC) samples were prepared by the same procedure described above. Furthermore, low, medium, high concentrations of QC samples of each component were 7.08, 141.50, 1132 ng/mL for butylidenephthalide; 5.22, 104.40, 835.20 ng/mL for ligustilide; 6.55, 131, 1048 ng/mL for senkyunolide A and 6.68, 667.50, 10,680 ng/mL for ferulic acid, respectively.

### 3.7. Plasma Sample Preparation

The collected plasma samples (100 µL) spiked with IS (10 µL) and methanol (10 µL) were vortex-mixed for 5 min. Subsequently, methanol of 300 µL was added for protein precipitation. The mixtures were vortexed for 5 min and then centrifuged at 13,000 rpm for 20 min. Finally, the supernatant was transferred to another clean centrifuge tube and centrifuged again in the same manner. The supernatant of 5 µL was injected into the UHPLC-Q-Exactive-Orbitrap/MS system for analysis.

### 3.8. Method Validation

#### 3.8.1. Specificity

The specificity of the method was investigated by comparing the chromatograms of blank plasma from six rats, blank plasma spiked with butylidenephthalide, ligustilide, senkyunolide A and ferulic acid at LLOQ and IS, and actual plasma samples after oral administration of Chuanxiong Rhizoma sample.

#### 3.8.2. Linearity and Lower Limit of Quantitation (LLOQ)

The standard curve was constructed by plotting the peak area ratio (y) of each analyte to IS (y) versus plasma concentrations (x) using a weighted least-square linear regression. The linear range was defined as the interval between the lowest and highest concentrations of the standard curve. In addition, the LLOQ was determined as the lowest concentration on the calibration curve, at which the acceptable precision (RSD) and accuracy (RE) cannot exceed 20% according to guidance. LLOQ could reflect the sensitivity of the method.

#### 3.8.3. Precision and Accuracy

The precision of each assay was evaluated through intra-day and inter-day precision by QC samples at low, medium, high concentrations levels respectively. It was expressed by the relative standard deviation (RSD). The intra-day precision and accuracy were determined by repeated analyses of six samples at each QC level during one day while the inter-day precision was determined by repeated analyses of six replicates of each QC sample on three consecutive days. The accuracy was calculated as follows: RE (%) = ((measuring value − nominal value)/nominal value) × 100%. The concentration of each sample was calculated by the regression equation of the latest calibration curve of each day. It is acceptable that RSD value is less than 15% and RE value is within ± 15%.

#### 3.8.4. Extraction Recovery and Matrix Effect

The extraction recovery was evaluated by comparing the peak areas of processed low, medium and high QC samples with standard solutions of the same concentration. The extraction recovery of IS was analyzed by comparing the peak area of extracted plasma samples at one concentration with standard solutions of the same concentration. The matrix effect was assessed by comparing the mean peak area of analyte spiked post-extraction with the mean area of a neat standard solution at equivalent concentration.

#### 3.8.5. Stability

The stability study of biological samples containing short-term, long-term, freeze-thaw and post-preparation stability could evaluate the influence of sample handling environment and sample storage environment on active component content. Six replicates of QC samples at three concentration levels were analyzed for all stability studies. Short-term stability was evaluated by keeping all QC samples at room temperature for 4 h before processing. Long-term stability was evaluated after all QC samples were stored at −20 °C for 60 days. Freeze-thaw stability of QC samples of each concentration was evaluated after three freeze (−20 °C)-thaw (room temperature) cycles. Post-preparation stability was to observe the stability of the extracted QC samples after waiting for 24 h in the autosampler at 4 °C. The concentration of each QC sample was calculated by the standard curve prepared freshly on the same day. The samples were considered stable when RSD of QC samples at three concentration levels was within ±15%.

### 3.9. Pharmacokinetic Study

Twelve rats were randomly divided into two groups (*n* = 6 per group), an orally administered liquid medicine of RCR and WCR respectively at the same dose of 24.01 g/kg. The blood samples (0.3 mL) from the suborbital vein of each rat were collected in a 1.5 mL heparinized centrifuge tube before dosing and at 0.033, 0.083, 0.16, 0.25, 0.5, 1, 2, 4, 8, 11, 24, 48 h after administration. The blood samples were immediately centrifuged at 13,000 rpm for 10 min and the plasma separated out were transferred to clean centrifuge tubes and stored at −20 °C until analysis.

### 3.10. Statistical Analysis

The pharmacokinetic parameters of the analytes were evaluated using Phoenix WinNonlin software (version 6.0, Pharsight Corporation, CA, USA). The mean plasma concentration-time curves were presented using Graphpad Prism (version 6.0, Graghpad Software, San Diego, CA, USA).

## 4. Conclusions

In this study, a sensitive and reliable UHPLC-Q-Exactive-Orbitrap/MS method was established and validated for the simultaneous quantification of butylidenephthalide, ligustilide, senkyunolide A and ferulic acid in rat plasma after oral administration of RCR and WCR for the first time. The superiority of this method lies in time and effort saving (simultaneous monitoring both positive and negative ions in one injection), high specificity and accuracy (exact molecular mass was used for quantitative analysis), and high sensitivity. The pharmacokinetic profiles of these components after oral administration of RCR and WCR were firstly compared to provides the scientific basis for the influence of wine processing the efficacy of Chuanxiong Rhizoma in vivo. We newly found that, even though the contents of butylidenephthalide, ligustilide, and ferulic acid in oral liquid medicine of WCR were higher than RCR (about four times higher), the AUC values (both AUC_0→t_ and AUC_0→∞_) and *C_max_* values were still decreased amazingly in WCR group. On the contrary, the V_d_ values of all compounds were increased in the WCR group by comparing it to the RCR group. These reminded us that the deep influence of processing with rice wine on Chuanxiong Rhizoma may not for increasing the blood concentrations of active compounds, but for promoting the blood circulation and increasing the distribution.

## Figures and Tables

**Table 1:** Mass spectrometric analysis parameters of four analytes and IS.

Analyte	T_R_	Molecular Formula	Ion Mode	Theoretical Mass (*m/z*)	Measured Mass (*m/z*)	Mass Error (ppm)
Butylidenephthalide	8.05	C_12_H_12_O_2_	[M + H]^+^	189.09100	189.09105	0.26
Ligustilide	8.38	C_12_H_14_O_2_	[M + H]^+^	191.10665	191.10664	0.05
Senkyunolide A	7.86	C_12_H_16_O_2_	[M + H]^+^	193.12230	193.12244	0.72
Ferulic acid	3.99	C_10_H_10_O_4_	[M − H]^−^	193.05063	193.04997	3.41
Calycosin-7-*O*-*β*-d-glucoside (IS)	4.05	C_22_H_22_O_10_	[M + H]^+^	447.12857	447.12817	0.89

**Table 2:** Precision, accuracy, extraction recovery and matrix effect of butylidenephthalide, ligustilide, senkyunolide A and ferulic acid in rat plasma. (*n* = 3 days and six replicates per day).

Component	Added (ng/mL)	Precision (RSD%)		Accuracy (RE%)	Extraction Recovery		Matrix Effect	
		Intraday	Interday		Mean (%)	RSD (%)	Mean (%)	RSD (%)
Butylidene-phthalide	7.08	8.49	10.97	4.77	106.38	2.22	93.82	9.06
	141.50	11.05	12.71	11.70	103.41	9.33	100.88	6.38
	1132.00	12.88	10.07	4.83	105.70	3.57	95.63	4.68
Ligustilide	5.22	6.94	11.39	1.87	92.61	3.68	98.13	5.00
	104.40	11.04	10.25	7.85	94.71	3.05	100.01	3.39
	835.20	9.49	11.44	−2.75	97.58	2.80	93.07	2.43
Senkyuno-lide A	6.55	5.17	7.40	6.54	101.54	2.76	92.29	3.38
	131.00	11.16	10.92	8.05	110.78	6.32	92.00	2.41
	1048.00	10.84	9.67	0.82	98.14	2.43	98.47	1.28
Ferulic acid	6.68	5.70	8.06	−8.14	109.05	12.39	105.63	1.34
	667.50	8.55	6.43	9.00	101.69	6.69	95.48	2.57
	10680.00	6.18	9.36	1.26	107.63	5.81	91.23	3.42

**Table 3:** Stability of butylidenephthalide, ligustilide, senkyunolide A and ferulic acid in rat plasma at different conditions (*n* = 6).

Component	Added (ng/mL)	Bench-Top Stability		Freeze-Thaw Stability		Post-Preparative Stability		Long-Term Stability	
		RSD (%)	RE (%)	RSD (%)	RE (%)	RSD (%)	RE (%)	RSD (%)	RE (%)
Butylidene-phthalide	7.08	3.84	1.88	3.36	−10.99	5.39	6.90	8.82	4.03
	141.50	5.23	11.97	3.55	6.03	9.24	2.22	6.50	−11.51
	1132.00	4.12	5.99	2.68	9.56	2.33	−5.45	8.93	6.65
Ligustilide	5.22	10.87	−4.49	7.63	2.45	4.22	6.32	6.56	−5.89
	104.40	11.70	5.17	9.25	1.98	9.25	4.70	7.24	11.82
	835.20	6.31	2.20	11.22	−3.59	2.85	−3.00	11.07	8.02
Senkyuno-lide A	6.55	9.48	5.21	3.35	7.70	10.64	−9.98	4.07	4.23
	131.00	4.01	9.44	7.38	11.86	3.58	−8.14	7.46	6.84
	1048.00	4.78	8.16	4.01	1.25	1.03	2.41	8.68	−5.75
Ferulic acid	6.68	2.21	7.26	6.30	−3.62	1.74	−6.55	1.74	−9.60
	667.50	2.87	10.16	5.32	6.02	6.79	−2.49	9.84	5.22
	10680.00	7.00	3.89	2.77	−2.01	0.57	7.42	6.75	3.92

**Table 4:** Pharmacokinetic parameters of butylidenephthalide, ligustilide, senkyunolide A and ferulic acid after oral administration of RCR and WCR at a dose of 24.01 g/kg (*n* = 6, mean ± SD).

Parameter	Butylidenephthalide		Ligustilide		Senkyunolide A		Ferulic Acid	
	RCR	WCR	RCR	WCR	RCR	WCR	RCR	WCR
AUC_0→t_ (h ng/mL)	743.30 ± 279.26	384.39 ± 90.92 *	4082.55 ± 201.34	3309.08 ± 578.87 *	1641.80 ± 438.09	803.64 ± 279.05 **	13,057.50 ± 12,329.37	4455.25 ± 1235.73
AUC_0→∞_ (h*ng/mL)	781.82 ± 251.72	401.58 ± 77.07 *	4112.55 ± 284.83	4043.32 ± 1481.63	1842.76 ± 500.73	860.81 ± 266.71 **	13,366.56 ± 12,420.74	4622.02 ± 1287.10
Cl (mL/h)	36.61 ± 16.33	234.35 ± 44.10 ***	128.76 ± 19.21	568.93 ± 184.86 **	392.87 ± 142.41	859.38 ± 319.21 *	24.08 ± 14.39	178.84 ± 54.46 **
*C_max_* (ng/mL)	288.43 ± 369.89	52.14 ± 16.63	289.42 ± 8.10	192.21 ± 8.48 ***	653.35 ± 580.97	151.03 ± 84.32	47,747.78 ± 51,447.40	9527.87 ± 6496.42
*t*_1/2_ (h)	11.41 ± 5.88	11.01 ± 3.75	8.50 ± 1.41	14.61 ± 7.56	13.44 ± 3.56	6.32 ± 1.72 **	8.57 ± 4.42	5.33 ± 2.97
MRT (h)	14.11 ± 6.54	13.94 ± 3.63	13.46 ± 1.74	21.94 ± 11.17	18.84 ± 4.24	9.86 ± 1.53 **	3.21 ± 1.66	4.05 ± 1.07
*T_max_* (h)	0.25 ± 0.14	0.27 ± 0.14	0.22 ± 0.05	0.30 ± 0.18	0.11 ± 0.09	0.18 ± 0.07	0.12 ± 0.09	0.06 ± 0.03
V_d_ (mL)	633.78 ± 424.90	3802.37 ± 1676.69 **	1579.29 ± 363.96	10,580.62 ± 2206.75 ***	7378.16 ± 2565.01	8275.68 ± 5216.70	283.95 ± 234.10	1519.30 ± 1321.97

Compared to RCR group, * *P* < 0.05, ** *P* < 0.01, *** *P* < 0.0001.
